# 800 Increased Incidence of Intraoperative Hypothermia in Burn Patients Administered Famotidine Preoperatively

**DOI:** 10.1093/jbcr/irae036.340

**Published:** 2024-04-17

**Authors:** Sai Pranathi Bingi, Taha Jilani, John Free, Alan Pang, John A Griswold

**Affiliations:** Texas Tech University Health Sciences Center School of Medicine, Lubbock, TX; Texas Tech University Health Sciences Center School of Medicine, Amarillo, TX; Texas Tech University Health Sciences Center, Lubbock, TX; Texas Tech University Health Sciences Center School of Medicine, Lubbock, TX; Texas Tech University Health Sciences Center School of Medicine, Amarillo, TX; Texas Tech University Health Sciences Center, Lubbock, TX; Texas Tech University Health Sciences Center School of Medicine, Lubbock, TX; Texas Tech University Health Sciences Center School of Medicine, Amarillo, TX; Texas Tech University Health Sciences Center, Lubbock, TX; Texas Tech University Health Sciences Center School of Medicine, Lubbock, TX; Texas Tech University Health Sciences Center School of Medicine, Amarillo, TX; Texas Tech University Health Sciences Center, Lubbock, TX; Texas Tech University Health Sciences Center School of Medicine, Lubbock, TX; Texas Tech University Health Sciences Center School of Medicine, Amarillo, TX; Texas Tech University Health Sciences Center, Lubbock, TX

## Abstract

**Introduction:**

Interoperative hypothermia is a surgical complication that can result in a variety of adverse intraoperative and postoperative outcomes such as cardiovascular events, infection, and hemorrhage. Burn patients are especially at risk of hypothermia due to prolonged surface exposure and impaired skin thermoregulation. The stress and metabolic demands caused by burn injuries further impair the body’s thermoregulatory responses. Famotidine, a widely used H2 receptor antagonist, is administered preoperatively to prevent stress ulcers in patients undergoing surgery with general anesthesia. Previous studies have shown that Famotidine can decrease core temperature through central thermoregulation. However, other studies provide conflicting results. As burn patients are especially at risk of interoperative hypothermia, it is important to study the role of famotidine in producing intraoperative hypothermia in these patients under general anesthesia.

**Methods:**

To answer this question, we conducted a retrospective study using the electronic medical records of Burn Intensive Care Unit patients. Patients were matched based on demographic criteria such as age, gender, body mass index (BMI), and race. We defined intraoperative hypothermia as a decrease in core body temperature below 36.5 degrees Celsius during a surgical procedure.

**Results:**

When comparing groups using a chi-squared test of independence, we found that patients who received famotidine preoperatively (n = 58) had a higher incidence of interoperative hypothermia versus those that did not receive famotidine preoperatively (n = 137). The statistical significance of this incidence was higher when excluding BMIs of greater than 30 (p = 0.002) versus greater than 35 (p = 0.009) or greater than 40 (p = 0.065).

**Conclusions:**

The observed relationship between patients who received famotidine preoperatively and interoperative hypothermia is stronger when excluding patients with a BMI greater than 30 versus excluding patients with a BMI greater than 40. This relationship may be due to the fact that those with higher BMIs are less likely to develop hypothermia, overcoming the physiological effect that famotidine creates.

**Applicability of Research to Practice:**

Given the relationship between thermoregulation and potential mortality, there is a clear motivation to reduce hypothermia in burn patients to improve patient outcomes. In fact, hypothermia increases the risk of infection, which is a major cause of mortality in burn patients. Since Famotidine was shown to increase the incidence of intraoperative hypothermia under general anesthesia, we suggest a safer option be explored.

